# Case Report: Convalescent Plasma Therapy Induced Anti-SARS-CoV-2 T Cell Expansion, NK Cell Maturation and Virus Clearance in a B Cell Deficient Patient After CD19 CAR T Cell Therapy

**DOI:** 10.3389/fimmu.2021.721738

**Published:** 2021-08-12

**Authors:** Berislav Bošnjak, Ivan Odak, Christiane Ritter, Klaus Stahl, Theresa Graalmann, Lars Steinbrück, Rainer Blasczyk, Christine S. Falk, Thomas F. Schulz, Hans Heinrich Wedemeyer, Markus Cornberg, Arnold Ganser, Reinhold Förster, Christian Koenecke

**Affiliations:** ^1^Institute of Immunology, Hannover Medical School, Hannover, Germany; ^2^Department of Gastroenterology, Hepatology and Endocrinology, Hannover Medical School, Hannover, Germany; ^3^Department of Rheumatology and Clinical Immunology, Hannover Medical School, Hannover, Germany; ^4^TWINCORE, Centre for Experimental and Clinical Infection Research, Hannover, Germany; ^5^Institute of Virology, Hannover Medical School, Hannover, Germany; ^6^Institute of Transfusion Medicine and Transplant Engineering, Hannover Medical School, Hannover, Germany; ^7^Institute of Transplantation Immunology, Hannover Medical School, Hannover, Germany; ^8^ German Center for Infection Research (DZIF), Partner Site, Hannover-Braunschweig, Germany; ^9^Cluster of Excellence RESIST (EXC 2155), Hannover Medical School, Hannover, Germany; ^10^Centre for Individualized Infection Medicine (CiiM) , Hannover, Germany; ^11^Department of Hematology, Hemostasis, Oncology and Stem Cell Transplantation, Hannover Medical School, Hannover, Germany

**Keywords:** convalescent plasma (CP), COVID – 19, SARS – CoV – 2, NK cell, T cell, CD19 CAR-T cell

## Abstract

Here, we described the case of a B cell-deficient patient after CD19 CAR-T cell therapy for refractory B cell Non-Hodgkin Lymphoma with protracted coronavirus disease 2019 (COVID-19). For weeks, this patient only inefficiently contained the virus while convalescent plasma transfusion correlated with virus clearance. Interestingly, following convalescent plasma therapy natural killer cells matured and virus-specific T cells expanded, presumably allowing virus clearance and recovery from the disease. Our findings, thus, suggest that convalescent plasma therapy can activate cellular immune responses to clear SARS-CoV-2 infections. If confirmed in larger clinical studies, these data could be of general importance for the treatment of COVID-19 patients.

## Introduction

The induction of antibodies binding to the receptor-binding domain (RBD) of the spike (S) protein of severe acute respiratory syndrome coronavirus 2 (SARS-CoV-2) has an important role in preventing virus infection and combating coronavirus disease 2019 (COVID-19) (1). In B cell deficient patients, who usually suffer from protracted COVID-19, convalescent plasma (CP) treatment proved to be helpful for controlling SARS-CoV-2 infection (2–5). However, the use of CP therapy in the treatment of immunoproficient patients with COVID-19 remains controversial. Although early meta-analyses indicated that prompt CP transfusion protects patients from lethal outcome (3), recent randomized trials could not confirm this finding (6).

Here, we describe the clinical course of a SARS-CoV-2 infection in a patient after CD19 CAR-T immunotherapy (CART) who had complete B cell depletion. Interestingly, the patient was able to contain the infection for five weeks but then developed aggravated symptoms leading to hospitalization. The symptoms rapidly resolved upon treatment with CP, which coincided with increase in SARS-CoV-2-specific T cell responses, natural killer (NK) cell maturation and decreased plasma levels of IL-6 and CXCL10. Overall, these data suggest an important role of SARS-CoV-2-specific antibodies in aiding endogenous NK and T cell responses to control SARS-CoV-2 infections.

## Case Description

A female patient received CD19 CART (Tisagenlecleucel) for the treatment of refractory diffuse large B-cell lymphoma on March 23, 2020, which led to complete remission of the lymphoma. Observed long-term side effects of CART were secondary hypogammaglobulinemia and prolonged pancytopenia. Hence, the patient received monthly intravenous immunoglobulin (IVIG), blood transfusions and intermittent G-CSF administration. IVIG treatment consisted of 10g Ig vena^©^, a commercially available pooled unspecific polyclonal human IgG serum (IgG1 62.1% IgG2 34.8%, IgG3 2.5%, IgG4 0.6%, IgA <50µg/mL), while the G-CSF (5µg/kg body weight/day) was administered intermittently in the months after CART when the neutrophil count was <500/µl.

**Figure 1:**
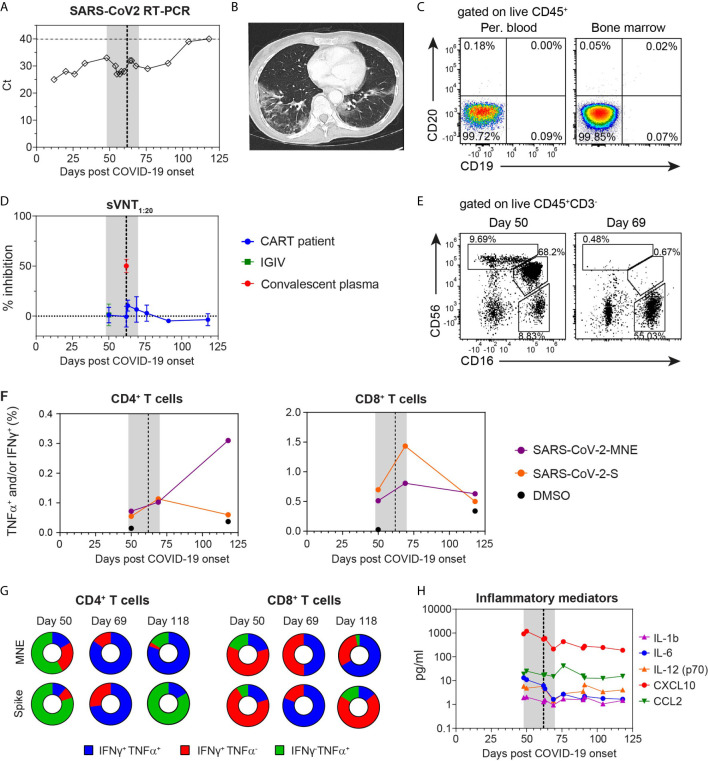
Protracted COVID-19 in a patient after CD19 CART resolved after CP therapy that coincided with increase in anti-SARS-CoV2 T cell-mediated response. **(A)** SARS-CoV-2 Ct values measured with RT-PCR. **(B)** Lung CT scan at day 51 post infection showing typical pattern of COVID-19 disease. **(C)** Complete B cell-deficiency in peripheral blood and bone marrow analyzed 10 months after CAR-T cell treatment shown by flow cytometry. **(D)** Neutralizing anti-SARS-CoV-2 antibody levels measured as inhibition of anti-SARS-CoV2 S-RBD with hACE2 at 1:20 plasma dilution by sVNT. **(E)** Blood NK cell show a CD16^+^CD56^-^ phenotype following CP therapy. **(F)** Total percentage of interferon gamma (IFN-γ) and/or tumor-necrosis factor alpha (TNF-α) producing CD8^+^ and CD4^+^ T cells following *ex vivo* stimulation with pooled spike (S)- or membrane, nucleocapsid, and envelope (MNE) peptide pools, gated as shown in **Supplementary Figure 1**. **(G)** Relative distribution of CD8^+^ and CD4^+^ T cells producing IFN-γ and/or TNF-α at time-points indicated. **(H)** Concentration of 5 selected of 27 measured inflammatory mediators in plasma. Depicted mediators have either markedly changed their concentrations post CP treatment. The concentrations of all tested cytokines are listed in **Supplementary Table 1**. **(A, D, F, H)** Shaded area indicates hospitalization; vertical dashed line indicates treatment with CP.

Eight months after CART, she tested positive for SARS-CoV-2 in an outpatient setting. Initial COVID-19 symptoms were mild, including fever, anosmia, ageusia and weight loss. The ongoing pancytopenia was aggravated, requiring platelet transfusions at a higher frequency. The patient was in home isolation and regularly seen by her local oncologist. Mild symptoms persisted over the next five weeks when she was (**Figure 1A** and **Table 1**). She was admitted to the specialized COVID-19 ward of Hannover Medical School 48 days post-symptom onset (PSO) due to aggravating clinical symptoms, in particular fever and cough. Due to low neutrophil counts on day of admittance, the patient received a single dose of G-CSF (5µg/kg bodyweight). SARS-CoV-2 infection was confirmed in house and viral genome sequencing (for details see Supplementary Materials) indicated that SARS-CoV-2 was of the B.1.36 lineage (**Supplementary Figure 1**). A chest CT scan showed COVID-19 pneumonia (**Figure 1B**). Of note, no oxygen support was required. The CT scan and bone marrow evaluation confirmed ongoing complete remission of the lymphoma. Bone marrow cytology showed a hypocellular marrow consistent with toxic damage. A complete B cell deficiency was demonstrated by flow cytometry on peripheral blood and bone marrow at the time of SARS-CoV-2 infection (**Figure 1C**). Therefore, the patient received (unspecific) IVIG treatment post hospitalization. At day 63 PSO, due to recurrent febrile episodes and undulating viral loads in nasopharyngeal swabs, the patient was also transfused with 295 ml of SARS-CoV-2 CP with neutralizing capacity of >90% and titer of >1:100 with according to SARS-CoV-2 S protein-pseudotyped-vesicular stomatitis virus vector-based neutralization assay (7, 8). Within one week of CP treatment viremia decreased, the patient’s condition rapidly improved and fever subsided. Resequencing of a PCR-positive respiratory sample taken 68 days PSO showed that the virus had acquired a V127I substitution in the N-terminal domain of the S protein (**Supplementary Figure 1**). The patient was discharged on day 70 PSO to home isolation and continuous outpatient control. Within one week after hospital discharge, the viral load in nasopharyngeal swabs increased. Nevertheless, the patient remained asymptomatic and did not receive any additional treatments. At 3 months PSO, pancytopenia resolved and viral load in nasopharyngeal swabs decreased and 4 months PSO, the patient tested negative for SARS-CoV-2 (**Figure 1A**) and was free of any COVID-19 symptoms.

**Table 1:** SARS-CoV2 Ct values, COVID-19 symptoms and hospitalization data.

Days post symptom onset	Ct	Result	Symptoms	Setting
0	NA	pos.	anosmia, ageusia, weight loss, cough, tricytopenia, fever	outpatient
12	25	pos.	anosmia, ageusia, weight loss, cough, tricytopenia	outpatient
20	28	pos.	anosmia, ageusia, weight loss, cough, tricytopenia	outpatient
26	27	pos.	anosmia, ageusia, weight loss, cough, tricytopenia	outpatient
33	31	pos.	anosmia, ageusia, weight loss, cough, tricytopenia	outpatient
48	33	pos.	anosmia, ageusia, cough, tricytopenia	inpatient
54	30	pos.	anosmia, ageusia, cough, tricytopenia	inpatient
55	27	pos.	anosmia, ageusia, cough, tricytopenia, fever	inpatient
57	27	pos.	anosmia, ageusia, cough, tricytopenia, fever	inpatient
58	28	pos.	anosmia, ageusia, cough, tricytopenia, fever	inpatient
60	28	pos.	anosmia, ageusia, cough, tricytopenia, fever	inpatient
64	32	pos.	anosmia, ageusia, cough, tricytopenia	inpatient
65	32	pos.	anosmia, ageusia, cough, tricytopenia	inpatient
68	30	pos.	anosmia, ageusia, cough, tricytopenia	inpatient
76	29	pos.	anosmia, ageusia, tricytopenia	outpatient
90	31	pos.	increasing platelets	outpatient
104	39	pos.	increasing platelets	outpatient
118	–	neg.	increasing platelets	outpatient

NA, not applicable.

## Methods

The institutional review board approved this study (No. 8610-BO-K-2019). Written informed consent was obtained from the patient for the publication of any potentially identifiable images or data included in this article. Neutralizing anti-SARS-CoV-2-S antibodies in plasma were determined by a surrogate virus neutralization test (sVNT) as described (7). Patient blood and bone marrow was analyzed using flow cytometry. For T cell reactivation, PBMCs were stimulated *ex vivo* with overlapping peptide pools from indicated SARS-CoV-2 proteins. Intracellular cytokine expression was analyzed afterwards. All details are described in Supplementary Materials.

## Results

As expected, no anti-SARS-CoV-2-RBD antibodies in the IVIG preparation or patient’s plasma pre-CP could be detected by sVNT (**Figure 1D**). However, in pre-CP blood of the patient we detected presence of CD3^−^CD56^−^CD16^+^ NK cells, a NK population that recently engaged target cells (9)(**Figure 1E**). Moreover, in blood we also detected CD8^+^ and CD4^+^ T cell responses specific to SARS-CoV-2 spike (S), or membrane (M), nucleocapsid (N) and envelope (E) proteins (**Figure 1F**). Interestingly, at this time point antigen-specific CD4^+^ T cells predominantly produced TNF-α, while CD8^+^ T cells predominantly produced IFN-γ (**Figure 1G** and **Supplementary Figure 2**). Together, these data indicated that the patient’s cellular immune response was sufficient to contain but inadequate to clear the infection.

Post-CP, the neutralizing antibodies were measurable in the patient’s plasma for a week, albeit at very low levels (**Figure 1D**). The clinical improvement post-CP was accompanied by a rapid decrease of plasma IL-6 and CXCL10 (**Figure 1H** and **Table 1**) as well as marked expansion of SARS-CoV-2 S- and MNE-specific CD8^+^ and CD4^+^ T cell populations (**Figure 1F** and **Supplementary Figure 2**). Importantly, a markedly increased proportion of the antigen-specific CD8^+^ and CD4^+^ T cells simultaneously produced TNF-α and IFN-γ, indicating their activation and/or maturation (**Figure 1G**). Post-CP we also observed a strongly increased frequency of recently activated CD16^+^CD56^-^ NK cells (**Figure 1E**). Together, these data suggest that the CP therapy, in addition to directly neutralizing SARS-CoV-2 virus particles might have also boosted virus-specific T cell as well as NK cell responses.

Interestingly, we detected a peak of inflammatory cytokines in plasma post hospital release (**Figure 1H** and **Supplementary Table 1**), suggesting a second wave of immune response activation. Of note, at this time we detected a higher percentage of anti-SARS-CoV-2 MNE-specific CD8^+^ and CD4^+^ T cells than anti-SARS-CoV2 S-specific T cell populations (**Figure 1F** and **Supplement Figure 2**). Moreover, the antigen-specific T cells again predominantly one cytokine, suggesting resolution of the acute immune response (**Figure 1G**).

## Discussion

Here, we described the case of a B cell deficient patient after CD19 CART with protracted COVID-19. For weeks, this patient only inefficiently contained the virus while CP transfusion correlated with virus clearance. Interestingly, increased anti-SARS-CoV-2 T cell and NK cell responses coincided with plasma transfusion, presumably also contributing to the patient’s recovery.

The mechanisms of CP action could be multifaceted. Besides neutralizing antibodies, CP contains other components that could block pro-inflammatory cytokines, reduce complement activation, or provide direct antiviral effects (10, 11). Nevertheless, the fact that clinical benefits of CP positively correlate with titers of neutralizing plasma antibodies (3, 10, 12) suggests that the main mode of CP action by providing supplementation with neutralizing antibodies. In line with this hypothesis, supplementation with anti-SARS-CoV-2 monoclonal neutralizing antibodies also provides direct anti-viral effects, reduces viral loads and restrains the infection, while at the same time allows precise neutralizing antibody dosing (13). Interestingly, our observation of coinciding activation of NK cells and SARS-CoV-2-specific T cells after plasma therapy suggests additional mechanisms that helped to finally clear the infection. CP contains also antibodies targeting different viral proteins that contribute to the activation of NK cells by inducing antibody-dependent cell cytotoxicity (ADCC) (14). Alternatively, these antibodies could kill infected cells by stimulating complement-dependent cytotoxicity (CDC) (15). Additionally, these antibodies could contribute to antibody-dependent cellular phagocytosis (ADCP) and increased antigen presentation to T cells, which is in line with reports that antibodies have essential complementary roles to CD8^+^ T cells in protection against viral infections (16, 17). In line with those reports, we observed markedly expanded CD8^+^ and CD4^+^ SARS-CoV-2-specific T cells following CP therapy. Similarly to the data from our patient, a previous case report of a SCID patient also showed that an initial CP treatment suppressed SARS-CoV-2 viremia and enabled a successful allogeneic stem cell transplantation (4). However, SARS-CoV-2 infection was cleared only after successfully restoring T and NK cell functionality (4). On the other hand, another patient who became infected with SARS-CoV-2 25 days after receiving CAR T cells targeting the B cell maturation antigen for the treatment of multiple myeloma, succumbed to COVID-19 despite receiving CP and multiple other treatments (18). Of note, this patient received CP therapy shortly after lympho-depleting immunotherapy and on a second occasion, CP therapy was combined with immunosuppressive treatment with dexamethasone (18). It seems possible, therefore, that a high dose steroid treatment might have suppressed beneficial effects of CP on anti-SARS-CoV-2 T cell activation. In line with this hypothesis are findings of no additional benefits of CP over placebo when applied together with corticosteroids (19).

Of note, unopposed by fully functional immune system, the SARS-CoV-2 infection persisted for several months allowing the time to virus to accumulate different mutations. Hence, our data support an important role for B cells in preventing COVID-19 and suggest introducing CP therapy is early in the treatment of COVID patients, with the aim of shortening the time of viral replication and thereby the risk of creating more dangerous variants. Finally, detailed profiling of immune responses after therapy indicated that in addition to direct anti-viral effects, anti-viral antibodies might, directly or indirectly, activate NK and T cells, which should be confirmed in further studies.

## Data Availability Statement

The original contributions presented in the study are included in the article/**Supplementary Material**. Further inquiries can be directed to the corresponding author.

## Ethics Statement

The studies involving human participants were reviewed and approved by Hannover Medical School review board approved this study (No. 8610-BO-K-2019). The patients/participants provided their written informed consent to participate in this study.

## Author Contributions

BB, IO, CR, CF, and LS performed experiments. KS, TG, MC, HW, AG, and CK were involved in the treatment of the patient. RB provided essential reagents. TS, RF, and CK supervised lab work. BB, RF, and CK designed the study and wrote the manuscript. All authors contributed to the article and approved the submitted version.

## Funding

This work was supported by Deutsche Forschungsgemeinschaft, (DFG, German research Foundation) Excellence Strategy EXC 2155 “RESIST” to RF and TS (Project ID39087428), by funds of the state of Lower Saxony (14-76103-184 CORONA-11/20) to RF and (14-76103-184 CORONA-12/20) to TS, by funds of the BMBF (“NaFoUniMedCovid19” FKZ: 01KX2021; Project B-FAST) to RF and Deutsche Forschungsgemeinschaft, Projektnummer 158989968 - SFB 900/3 (Projects B1 to RF, C1 to TS, and B8 to CK), DZIF TTU-IICH 07-808 to CF.

## Conflict of Interest

The authors declare that the research was conducted in the absence of any commercial or financial relationships that could be construed as a potential conflict of interest.

## Publisher’s Note

All claims expressed in this article are solely those of the authors and do not necessarily represent those of their affiliated organizations, or those of the publisher, the editors and the reviewers. Any product that may be evaluated in this article, or claim that may be made by its manufacturer, is not guaranteed or endorsed by the publisher.
