# Supplementary material for: Case Report: Convalescent Plasma Therapy Induced Anti-SARS-CoV-2 T Cell Expansion, NK Cell Maturation and Virus Clearance in a B Cell Deficient Patient After CD19 CAR T Cell Therapy
Source: Front Immunol. 2021 Aug 12;12:721738. doi: 10.3389/fimmu.2021.721738 (PMC8387963; doi:10.3389/fimmu.2021.721738)
Supplement: Supplementary file 1 [file DataSheet_1.docx]

**Supplemental material and methods**

*CT scan*

The CT examination was performed in the Radiology Department of Hannover Medical School with a 16 row Multidetector Computed Tomography (MDCT, Lightspeed 16, GE Healthcare, Milwaukee, WI, USA). CT data was acquired volumetrically using a standard dose protocol with 120 kV and 100 mAs. CT data was reconstructed with a slice collimation of 1.25 mm and an interval of 1 mm. Intravenous contrast medium was used.

*SARS-CoV-2 protein peptide pools*

Overlapping peptides, each 15 aa long with 10 aa overlap, spanning the whole length of SARS-CoV2-S (total 253 peptides), -M (43 peptides), -N (82 peptides) or –E (12 peptides; peptide no 4 could not be synthesized) were used for T cell re-stimulation. Peptides were synthesized at >95% purity (GeneScript). All lyophilized peptides were reconstituted at a stock concentration of 50 mg/mL in DMSO (Sigma-Aldrich) except for 9 SARS-CoV2-S overlapping peptides (number 24, 190, 191, 225, 226, 234, 244, 245 and 246), 2 for SARS-CoV2-M (number 15 and 16), 1 for SARS-CoV2-N (number 61) and all 12 SARS-CoV2-E peptides that were dissolved at 25 mg/mL due to solubility issues. All peptides were stored at -80°C before use.

*T cell re-stimulation assay*

After isolation through a Ficoll gradient, PBMCs were re-suspended in complete RPMI medium [RPMI 1640 (Gibco) supplemented with 10% FBS (GE Healthcare Life Sciences, Logan, UT), 1mM sodium pyruvate, 50 µM β-mercaptoethanol, 1% streptomycin/penicillin (all Gibco)] at concentration of 20 x 10^6^ cells/ml and mixed 1:1 with S-protein or mixture of M-, N- and E-protein peptide pools. Peptide pools were dissolved in complete RPMI containing brefeldin A (Sigma-Aldrich) at final concentration of 10 µg/ml, while the final concentration of each peptide was 2 µg (~1.2 nmol)/ml, except for SARS-CoV2-S peptides number 24, 190, 191, 225, 226, 234, 244, 245 and 246, SARS-CoV2-M peptides 15 and 16, and SARS-CoV2-N peptide 61, which were used at final concentration of 1 µg/ml due to solubility issues. The maximal total amount of DMSO in the final cells suspension was ≤5 % that was also used without any peptides as negative control. As internal positive control, cells were stimulated with Phorbol-12-myristate-13-acetate (PMA; Calbiochem) and ionomycin (Invitrogen) at final concentration of 50 ng/mL and 1500 ng/mL, respectively. After 12-16 hr incubation at 37°C, 5% CO2, cells were collected, washed and resuspended in PBS. Samples were stained with anti-CD3-AF532 (UCHT1; #58-0038-42; Invitrogen), anti-CD4-BUV563 (SK3; #612913; BD Biosciences), anti-CD8-SparkBlue 550 (SK1; #344760; Biolegend) and Zombie NIR™ Fixable Viability Kit (#423106, BioLegend) for 20 minutes at 37°C. Following first incubation, cells were washed and then fixed and permeabilised (#554714, BD Biosciences) according to the manufacturers’ protocol. Samples were stained using, anti-IFN-PE-Cy7 (4S.B3, #502528, Biolegend) and anti-TNF-AF700 (Mab11; #561023; BD Biosciences) for 30 minutes on RT. After two washes, cells were acquired on Cytek Aurora spectral flow cytometer (Cytek) equipped with five lasers operating on 355nm, 405nm, 488nm, 561nm and 640nm. All flow cytometry data was analyzed using FCS Express V7 (Denovo).

*Spectral flow cytometry for leukocyte populations*

Whole blood was subject to erythrolysis in 0.83% ammonium chloride solution. Cells were further washed, re-suspended in PBS and stained with anti-CD45RA-BUV395 (HI100; #740298; BD Biosciences), anti-CD45RO-Pacific Blue (UCHL1; #304216; Biolegend), anti-CD3-AF532 (UCHT1; #58-0038-42; Invitrogen), anti-CD16-BUV496 (3G8; #612944, BD Biosciences), anti-CD56-BUV737 (NCAM16.2; #612766, BD Biosciences) and Zombie NIR™ Fixable Viability Kit (#423106, BioLegend) for 20 mins on RT. After one wash, samples were analysed by spectral flow cytometry.

*Cytokine, chemokine and growth factor detection*

Cytokine, chemokine and growth factor concentrations in plasma were quantified by the Luminex-based multiplex technique according to the manufacturer’s instructions (Bio-Rad, USA). The human 27-plex (M500KCAF0Y, Bio-Rad, Hercules USA) was used with 50 µl plasma, 1+1 diluted with sample diluent. Standard curves and concentrations were calculated with Bio-Plex Manager 6.2, the lower detection limit of all proteins was 1-5 pg/ml (1).

*Sequencing of SARS-CoV2 genome*

A quantitative SARS-CoV-2 RT-PCR was carried out as described earlier (2). To determine the complete sequence of SARS-CoV-2, RNA was extracted from patient samples using the Zymo QickViral RNA kit. 16 µl eluted RNA were reverse transcribed using 4 µl NEB Lunascript Supermix, following the NEB protocol. The sequencing library was prepared using the NEBnext ARTIC SARS-CoV-2 library preparation kit, 35 amplification cycles using the ARTIC primer panel and Q5 polymerase mastermix. Libraries were indexed using NEBnext Multiplex Oligos for Illumina, unique dual index primer pairs, 8nt Index i5 and i7, and 5 amplification cycles. Indexed libraries were sequenced on an Illumina MiSeq instrument. Analysis of the generated reads involved read trimming with fastp (adaptor and quality trimming, Q20), followed byread filtering against human genome hg19 using bowtie2 and read extraction using samtools and bam2fastq, then normalization/de-spiking using bbnorm.sh (cov 100), denovo assembly using coronaSPAdes (rna SPAdes with a set of Hidden Markov Models especially for SARS-CoV-2) and 3 rounds of iterative remapping and consensus correction using bbnorm.sh, bowtie2, samtools and pilon (correction coverage chosen by user, mostly 2000). This was followed by annotation and alignment in Geneious, and lineage determination using a homemade script to compare the assembled sequence to the pangolin database.

**Supplemental references**

1. Halama N, Zoernig I, Berthel A, Kahlert C, Klupp F, Suarez-Carmona M, Suetterlin T, Brand K, Krauss J, Lasitschka F, et al. Tumoral Immune Cell Exploitation in Colorectal Cancer Metastases Can Be Targeted Effectively by Anti-CCR5 Therapy in Cancer Patients. *Cancer Cell* (2016) **29**:587–601. doi:10.1016/j.ccell.2016.03.005

2. Cordes AK, Heim A. Rapid random access detection of the novel SARS-coronavirus-2 (SARS-CoV-2, previously 2019-nCoV) using an open access protocol for the Panther Fusion. *J Clin Virol* (2020) **125**: doi:10.1016/j.jcv.2020.104305

**Supplemental Figures**


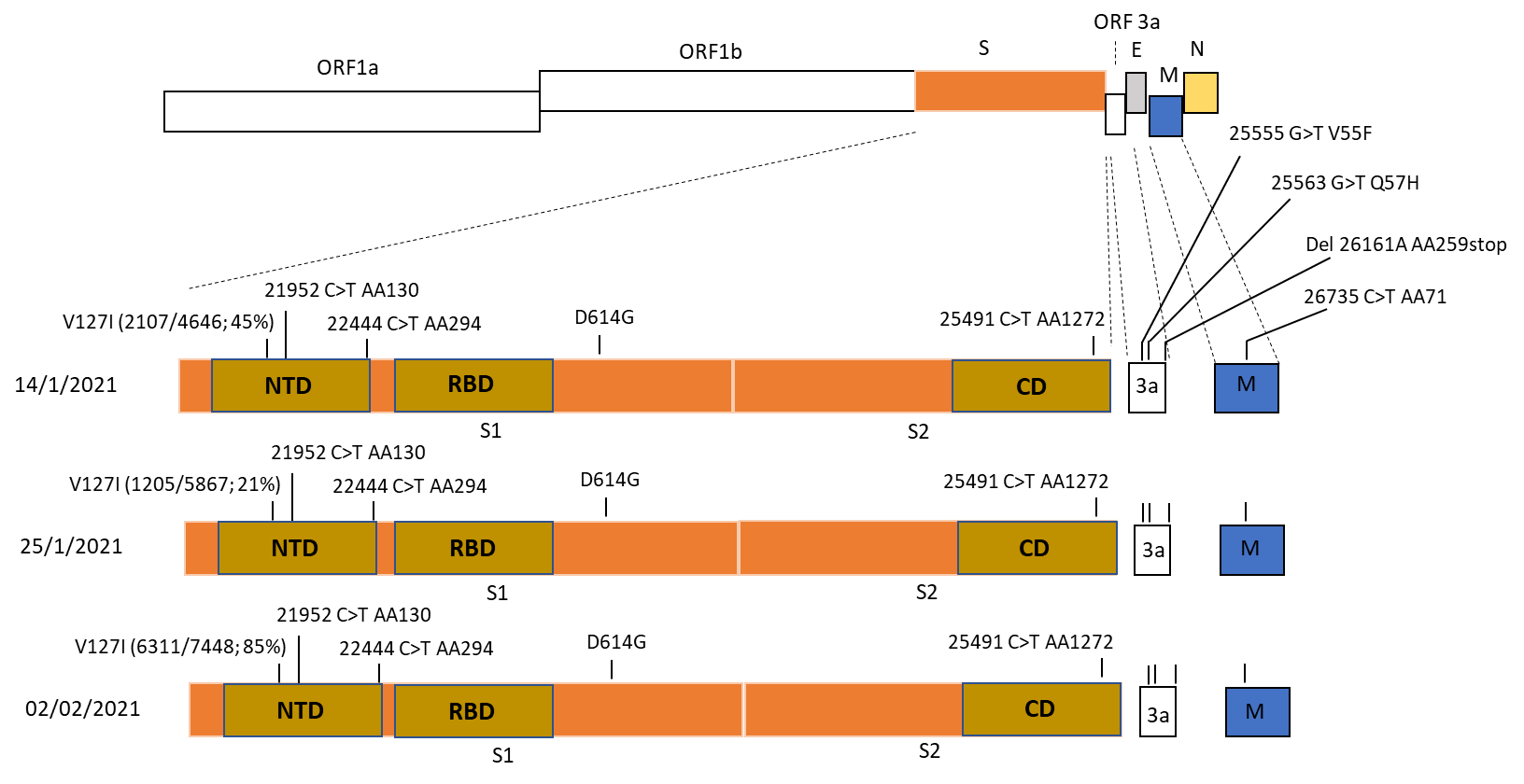


**Supplemental Figure 1.** Simplified diagram of the SARS-CoV-2 genome with the mutations found by NGS at the three time points. In position 127 of the S protein, a valine was gradually replaced with an isoleucine over the course of three weeks. The numbers in brackets behind the V127I mutation indicate the number of NGS reads showing this mutation/total number of reads at this position and the percentage of reads showing this mutation. All other mutations were “fixed”, i.e. have not changed over time. Abbreviations: RBD: receptor binding domain; NTD: N-terminal domain; CD cytoplasmic domain


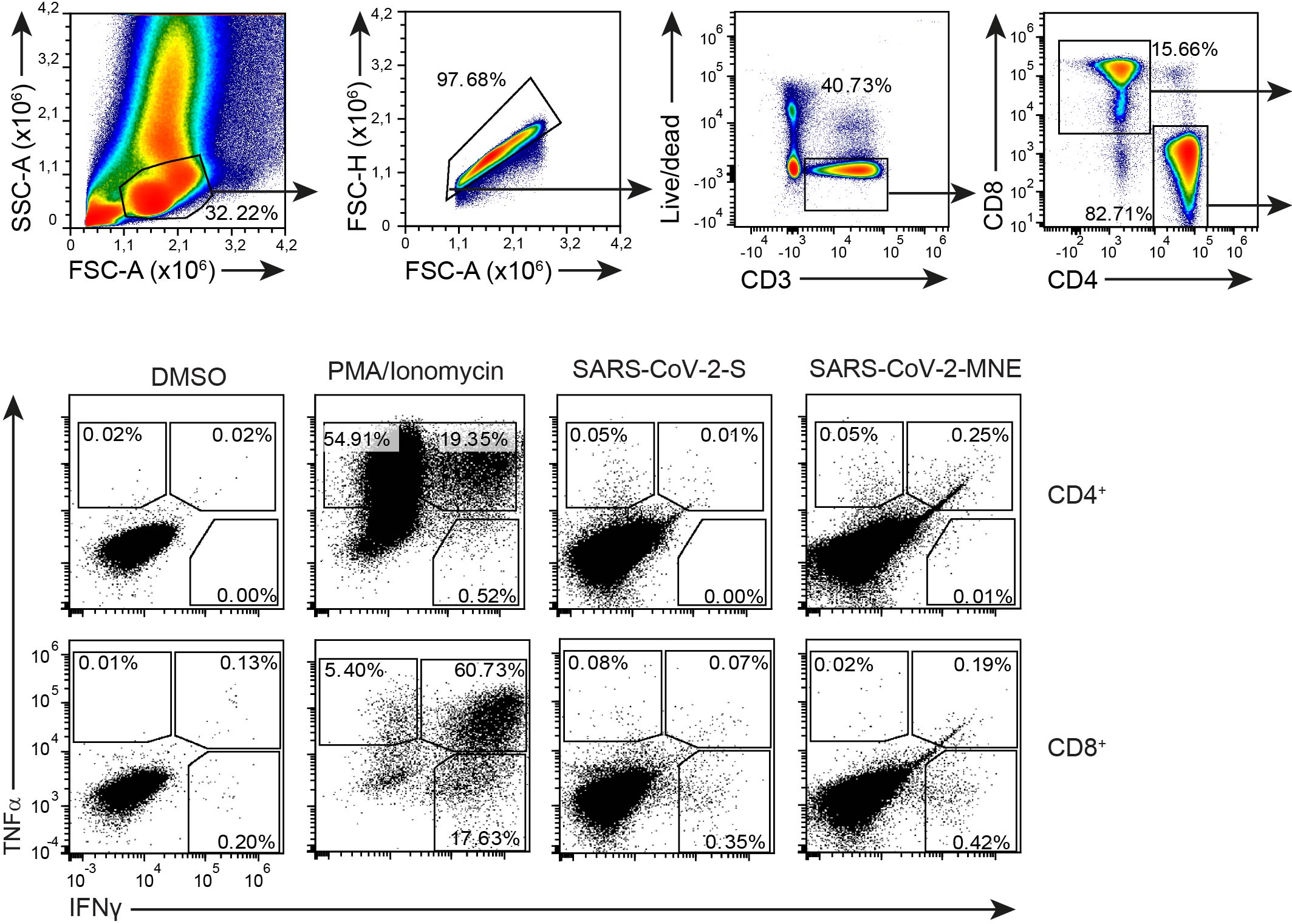


**Supplemental Figure 2.** Example of gating strategy for cytokine measurement in T cells after re-stimulation with DMSO, phorbol myristate acetate (PMA)/Ionomycin, or 15 amino acid-long, 10-amino acid overlapping peptide covering whole length SARS-CoV-2-Spike (S) or .Membrane (M), Nucleocapisd (N) and Envelope (E) proteins. Samples shown were from day 118 post SARS-CoV-2 infection.

**Supplemental Table 1.** Concentrations of inflammatory mediators in plasma (pg/ml) at various time-points post SARS-CoV2 infection measured by Luminex technology. BDL – below detection limit; ** indicates G-CSF treatment (5 µg/kg).

|  | **Days post SARS-CoV2 infection** | | | | | | | | | |
| --- | --- | --- | --- | --- | --- | --- | --- | --- | --- | --- |
| **Mediator** | **48**** | **50** | **62** | **63** | **69** | **76** | **90** | **91** | **104** | **118** |
| **IL-1b** | 1.93 | 2.04 | 1.3 | 1.53 | 0.95 | 1.7 | 1.59 | 2.04 | 1.06 | 1.47 |
| **IL-1RA** | 145.4 | 241.19 | 135.15 | 124.72 | 91.66 | 147.28 | 102.81 | 115.28 | 79.32 | 97.89 |
| **IL-2** | 8.33 | 8.95 | 8.18 | 8.64 | 5.38 | 9.42 | 8.49 | 10.35 | 7.86 | 6.93 |
| **IL-4** | 1.67 | 1.88 | 1.86 | 1.97 | 1.72 | 3.4 | 2.17 | 2.46 | 1.76 | 2.31 |
| **IL-5** | 15.64 | 20.43 | 19.65 | 13.98 | 3.71 | 16.46 | 17.27 | 32.26 | 18.07 | 17.27 |
| **IL-6** | 13.12 | 10.96 | 6.18 | 4.59 | 1.64 | 2.7 | 1.61 | 2.19 | 1.79 | 1.67 |
| **IL-7** | 20.14 | 19.38 | 22.03 | 23.16 | 22.53 | 39.39 | 29.92 | 31.42 | 22.15 | 29.05 |
| **IL-8** | 12.97 | 8.41 | 6.46 | 5.87 | 3.03 | 10.94 | 6.17 | 8.12 | 8.31 | 6.07 |
| **IL-9** | 23.92 | 20.46 | 19.88 | 18.14 | 9.22 | 21.91 | 20.46 | 25.07 | 23.35 | 19.88 |
| **IL-10** | 5.93 | 7.77 | 9 | 8.69 | 4.41 | 9 | 6.54 | 9.62 | 6.85 | 8.38 |
| **IL-12(p70)** | 5.78 | 4.86 | 5.78 | 4.47 | 1.4 | 2.84 | 3.52 | 6.68 | 3.39 | 4.06 |
| **IL-13** | 4.67 | 5.27 | 4.71 | 5.52 | 4.23 | 7.21 | 5.31 | 6.19 | 3.88 | 4.93 |
| **IL-15** | 18.62 | 29.98 | 17.7 | 19.54 | 4.39 | 20.45 | 23.12 | 31.64 | 21.35 | 21.35 |
| **IL-17** | 4.47 | 5.19 | 4.29 | 4.47 | 3.22 | 5.99 | 5.19 | 7.42 | 4.83 | 5.81 |
| **CCL11** | 27.69 | 36.03 | 25.74 | 32.43 | 32.83 | 98.46 | 58.47 | 63.76 | 49.57 | 56.09 |
| **FGFβ** | 8.79 | 10.43 | 9.62 | 10.27 | 7.93 | 11.85 | 11.54 | 12.46 | 9.95 | 10.43 |
| **G-CSF** | 70.57** | 2572.9 | 2498.67 | 1681.81 | 159.52 | 85.39 | 68.33 | 75.5 | 58.07 | 62.08 |
| **GM-CSF** | 2.29 | 1.99 | 1.78 | 1.73 | 0.35 | 1.89 | 1.78 | 2.58 | 1.57 | 1.68 |
| **IFN-γ** | BDL | BDL | BDL | BDL | BDL | BDL | BDL | BDL | BDL | BDL |
| **CXCL10** | 914.68 | 1178.64 | 548.69 | 590.5 | 212.85 | 432.43 | 233.06 | 278.07 | 245.26 | 188.12 |
| **CCL2** | 18.81 | 25.05 | 16.85 | 17.73 | 14.84 | 41.81 | 15.88 | 12.87 | 12.43 | 15.29 |
| **CCL3** | 2.48 | 3.24 | 3.2 | 3.17 | 2.69 | 3.1 | 2.3 | 2.51 | 2.25 | 2.18 |
| **PDGF-bb** | 24.85 | 73.03 | 24.85 | 23.7 | 9 | 22.56 | 63.51 | 96.43 | 126.1 | 32.94 |
| **CCL9** | 15.16 | 13.47 | 13.53 | 13.42 | 9.75 | 18.57 | 17.36 | 18.84 | 19.33 | 16.18 |
| **CCL5** | 420.45 | 923.28 | 192.29 | 121.22 | 118.75 | 550.58 | 971.83 | 1061.87 | 1190.32 | 713.2 |
| **TNF-α** | 14.7 | 18.05 | 16.47 | 14 | 9.36 | 19.45 | 14.7 | 17.52 | 15.06 | 12.93 |
| **VEGF** | 18.5 | 18.06 | 13.99 | 14.45 | 2.32 | 17.18 | 15.38 | 19.79 | 15.38 | 18.5 |
